# Slope-reducing tibial osteotomy yields better posterior horn healing performance of longitudinal and bucket-handle tears of medial meniscus in patients undergoing ACL reconstruction: a minimum 2-year follow-up study with second-look arthroscopy

**DOI:** 10.1186/s13018-026-07070-1

**Published:** 2026-06-30

**Authors:** Tong Zheng, Daofeng Wang, Guanyang Song, Hui Zhang

**Affiliations:** 1 Sports Medicine Service, Beijing Jishuitan Hospital, Capital Medical University, No. 31, Xinjiekou East Street, Beijing, 100035 China; 2 Beijing Research Institute of Traumatology and Orthopaedics, No. 31, Xinjiekou East Street, Beijing, 100035 China

**Keywords:** ACL reconstruction, Slope-reducing tibial osteotomy, Meniscus posterior horn tears, Healing rate, Graft laxity

## Abstract

**Purpose:**

To assess the effect of additional slope-reducing tibial osteotomy (SRTO) on the healing rate of repaired meniscus posterior horn tears (MPHTs) in ACL-injured patients with steep PTS and excessive anterior tibial subluxation (ATS).

**Methods:**

This retrospective study evaluated patients undergoing primary ACLR between 2016 and 2023 who met three inclusion criteria: PTS ≥ 15°, ATS≥6 mm, and repaired longitudinal and bucket-handle tears involving the meniscus posterior horn. Participants were stratified into an osteotomy group (ACLR + SRTO, *n* = 20) and a control group (ACLR alone, *n* = 48), with both cohorts maintaining ≥ 2-year follow-up. The primary endpoint compared meniscal horn healing rates between groups. Second, the associations between meniscal healing status and graft outcomes, as well as postoperative knee stability, were investigated.

**Results:**

Both groups were comparable in terms of age, gender, side, BMI, PTS, graft diameter, time from injury to surgery, ATS, KT side-to-side differences, and pivot-shift grade (*all P* > 0.05). In the osteotomy group, 17 medial meniscus tears were identified (11 longitudinal and 6 bucket-handle tears). At the final follow-up, partial healing of medial MPHTs was achieved in 1 longitudinal tear, resulting in an overall healing rate of 94.1% (16/17). The control group presented with 34 meniscal tears (23 longitudinal + 11 bucket-handle tears). The final follow-up assessment revealed partial healing of medial MPHTs in 8 longitudinal and 2 bucket-handle tears, yielding a significantly lower overall healing rate of 70.6% (24/34) compared with the osteotomy group (*P* = 0.036). For lateral meniscus tears, the osteotomy group demonstrated partial healing of lateral MPHTs in 1 of 10 cases, compared with 5 of 33 cases in the control group, with no significant difference in overall healing rates between groups (90% vs. 84.8%, *P* = 0.680). In addition, partial healing of medial MPHTs was associated with residual graft laxity (50% vs. 16.7%, *p* = 0.045) compared to those with complete healing.

**Conclusion:**

For ACL-injured patients with steep PTS (≥ 15°) and excessive ATS (≥ 6 mm), the repaired medial MPHTs may benefit from the additional SRTO. In addition, incomplete healing of the posterior horn of the medial meniscus may be associated with residual ACL graft laxity.

**Clinical relevance:**

An additional slope-reducing tibial osteotomy may be indicated to facilitate medial meniscal healing in ACL injuries with excessive PTS and a concomitant posterior meniscal horn tear.

**Level of evidence:**

Cohort study III.

## Introduction

Previous studies have reported that meniscal horn tears occur in 18% of chronic anterior cruciate ligament (ACL)-deficient knees and 12% of acute ACL injuries [1, 2]. Bucket-handle and longitudinal meniscal tears, typically beginning at the posterior horn and progressing anteriorly, usually require suture repair [3, 4]. Postoperative healing at the posterior horn is a critical determinant of clinical success. Currently, numerous risk factors, such as age, higher BMI, and female sex, have been clinically validated to correlate with posterior meniscal horn tears [5, 6]. Although these factors are non-modifiable through surgical intervention, they play a crucial role in perioperative patient education and rehabilitation. In addition to clinical risk factors, recent studies have identified various anatomic risk factors for posterior meniscus horn tears and postoperative reinjuries, including but not limited to varus malalignment and increased posterior tibial slope (PTS) [7–9]. Cadaveric and biomechanical studies also found that an increased PTS affects compression and anterior shear forces at the posterior meniscus horn [10, 11].

What benefits can slope correction surgery provide for patients with steep PTS? Satisfactory functional outcomes and objective knee stability have led clinicians to gradually accept the combined procedures in ACL revision cases [12–17]. In these studies, slope correction was found to improve graft remodeling conditions and long-term outcomes, control anterior tibial translation, and enhance knee stability. Recent evidence also suggests that SRTO effectively reduces anterior tibial translation and improves graft outcomes in primary ACL-injured patients, with clinical benefits correlating with the degree of slope correction [18]. However, few studies have analyzed the effect of combined slope correction surgery on meniscal healing performance.

Therefore, the purpose of this study was to assess the effect of combined SRTO and primary ACL reconstruction (ACLR) on the healing rate of repaired longitudinal and bucket-handle tears in the posterior horn of the meniscus in patients with steep PTS. It was hypothesized that patients undergoing combined procedures would demonstrate better healing performance of MPHTs compared to those receiving ACLR alone.

## Methods

### Study design

This study was approved by the Ethics Committee of Beijing Jishuitan Hospital, Capital Medical University, and informed consent was obtained from the patients. A continuous sample of patients with a clinically diagnosed ACL injury who underwent primary ACLR in our center from June 2016 to January 2023 was retrospectively reviewed. To ensure a minimum follow-up of 2 years, the patients were advised to postpone internal fixation removal until 2 years after surgery. This study adheres to the STROBE checklist [19].

### Patient selection

**Fig. 1:**
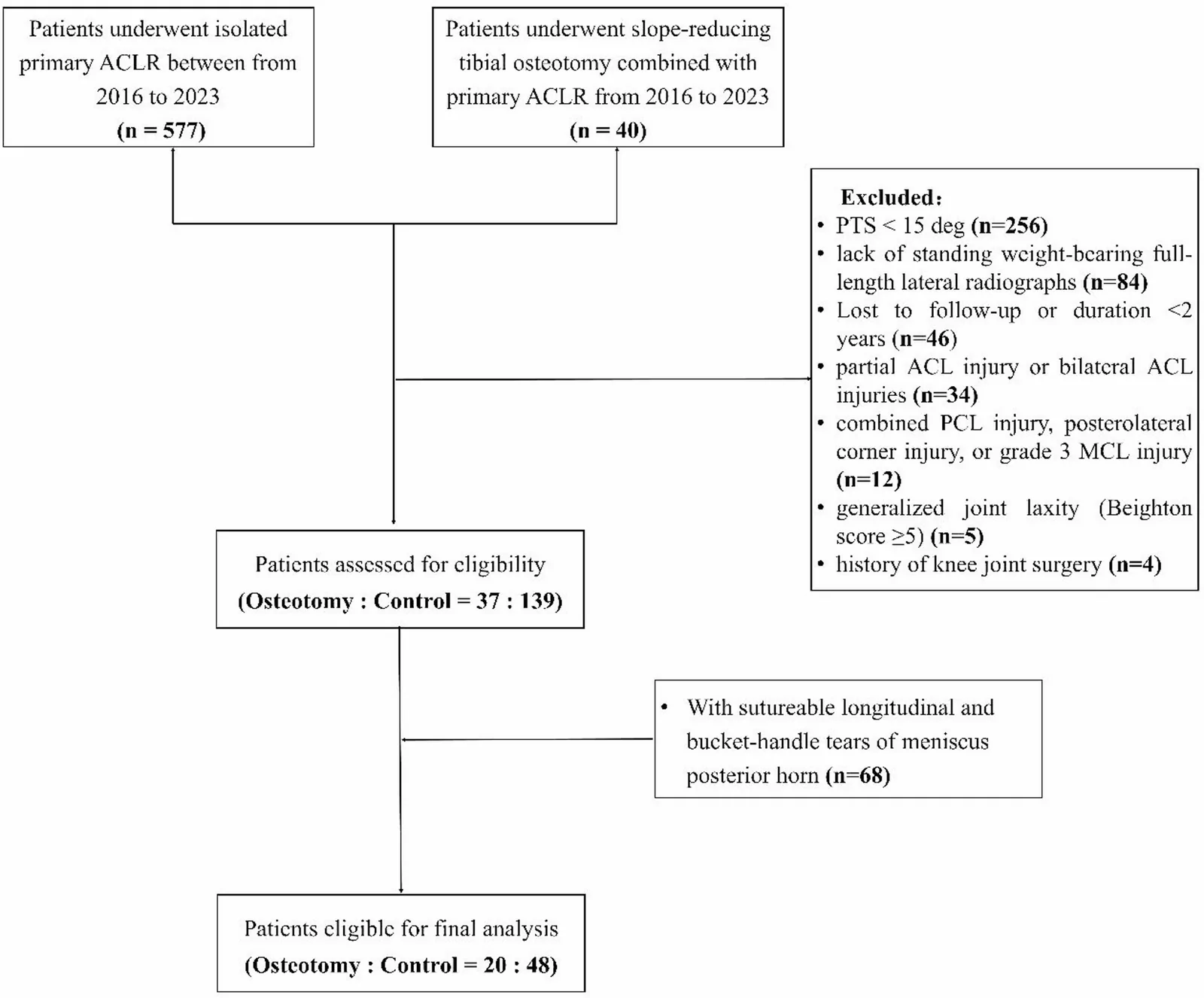
Flowchart of patient selection

ACL injury patients with (1) concomitant steep PTS (≥ 15°) and excessive ATS (≥ 6 mm); (2) the presence of longitudinal and bucket-handle tears involving the meniscus posterior horn receiving primary suture repair were included. For patients with concomitant steep PTS (≥ 15°) and excessive ATS (≥ 6 mm), we performed slope-reducing tibial osteotomy in combination with primary ACL reconstruction to improve the mechanical environment of the ACL graft. Most injuries were non-contact and occurred during sports activities. Exclusion criteria were as follows: (1) intact meniscus; (2) no concomitant longitudinal and bucket-handle tears of meniscus posterior horn; (3) presence of meniscus tears but without suture repair; (4) further exclusion criteria were: partial ACL injury, bilateral ACL injury, combined posterior cruciate ligament injury or grade 3 medial collateral ligament injury, preoperative hyperextension > 10° of the injured knee, mild to severe coronal-plane malalignment (varus/valgus > 5°) of the injured lower extremity, history of surgery to the lower extremities, and final follow-up < 2 years. The flowchart of patient selection is shown in Fig. 1.

It should be emphasized that all patients in this study underwent a second surgery at the final follow-up for the removal of tibial cortical screws. Patients who did not return for hardware removal were not considered for inclusion. Second-look arthroscopy was routinely performed during this second surgery.

### Grouping

Patients were divided into an osteotomy group and a control group based on whether they opted to undergo the osteotomy procedure. Two group analyses were designed: (1) primary group: patients were divided into the osteotomy group and the non-osteotomy group based on whether concomitant osteotomy was performed. This grouping was designed to compare posterior meniscal horn healing rates between two groups. (2) secondary group (subgroup analysis): patients were stratified into two groups according to the healing status of the repaired meniscus posterior horn. This stratification was designed to investigate the association between meniscal healing status and both graft outcomes and knee stability.

### Posterior tibial slope

**Fig. 2:**
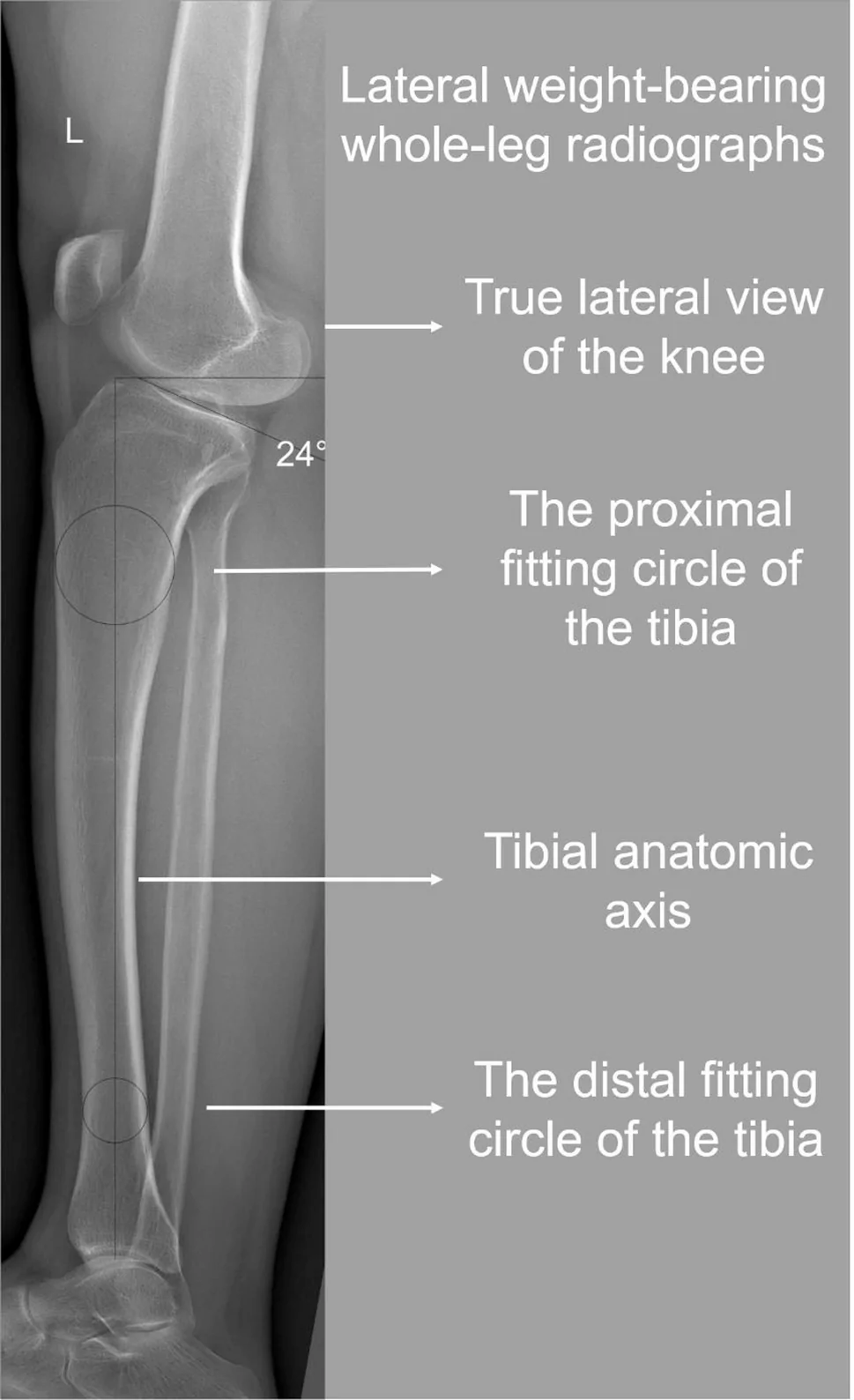
Measurement of the PTS [18]. On true lateral radiographs of the lower limb, fitting circles are made at the proximal and distal cortices of the tibia to determine the TAA. The angle between the tangent to the medial tibial plateau and the line perpendicular to the TAA is defined as the PTS

Whole-leg weight-bearing radiographs (EOS Imaging) were taken preoperatively for all patients. On a true lateral knee view with fluoroscopic condylar superimposition, the posterior tibial slope (PTS) was measured as the angle between the medial tibial plateau tangent and a line perpendicular to the tibial anatomic axis (TAA) (Fig. 2), following previously described method [18].

### Intervention

All procedures were performed by a single senior orthopedic surgeon **(H.Z)**. Both patient groups underwent ACLR with concomitant meniscus posterior horn repair. The osteotomy group received an additional slope-reducing tibial osteotomy. Detailed surgical techniques followed the method described by Wang et al. [18]. In brief, the standardized procedure included:

### ACL reconstruction

The semitendinosus and gracilis tendons were harvested and prepared as an 8-strand graft for ACL reconstruction [20]. Under arthroscopic guidance, the tibial tunnel was created using a standard ACL tibial guide. Femoral tunnel preparation was performed through an anteromedial portal with the knee flexed > 120°, utilizing intraoperative fluoroscopy for precise positioning. Finally, the ACL graft was secured using the suspensory fixation technique [18].

### Slope-reducing tibial osteotomy

**Fig. 3:**
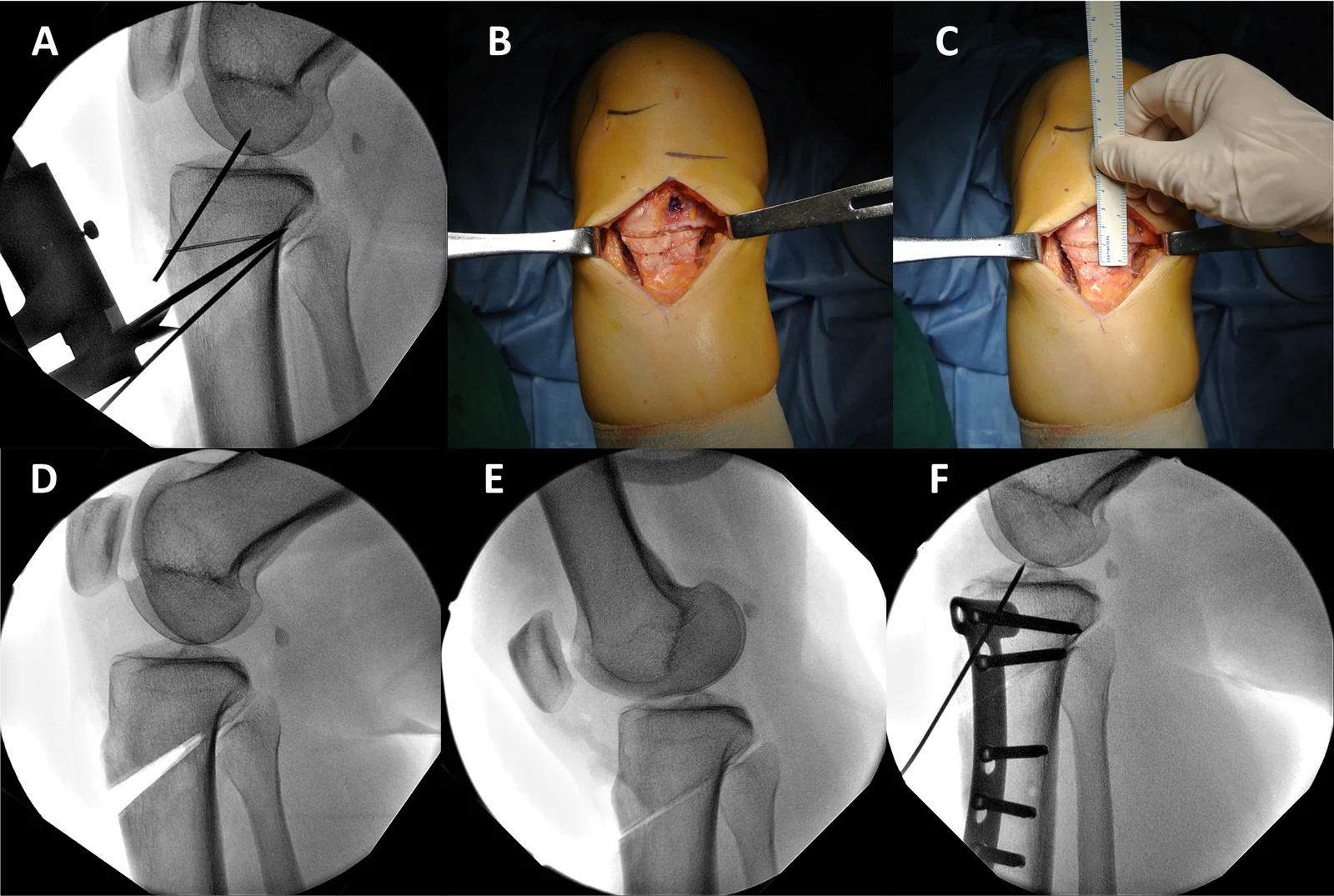
Slope-reducing tibial osteotomy (SRTO). **A** The upper and lower osteotomy planes were determined under fluoroscopy based on preoperative planning; **B** Perform the osteotomy using an oscillating saw; **C** The wedge-shaped bone segment was 9 mm in thickness; **D** Preserve the posterior cortex intact; **E** The osteotomy site was gradually closed by knee extension and compression; **F** Fix the osteotomy site using plate and screw system

A biplanar osteotomy was performed about 2 cm distal to the tibial tubercle. Under fluoroscopic guidance, biplanar osteotomy planes were determined according to preoperative planning (typically correcting 8–10°). The wedge-shaped bone fragment was removed using an oscillating saw, preserving a posterior cortical hinge. Gradual knee extension with simultaneous compression achieved complete osteotomy closure, followed by TomoFix system (Synthes, America) fixation (Fig. 3).

### Arthroscopic meniscus repair

For medial meniscal longitudinal or bucket-handle tears involving the posterior horn, the inside-out repair technique using a non-absorbable braided suture with the needle (ConMed Corporation, USA) was the preferred approach. For lateral meniscal tears involving the posterior horn, the all-inside repair technique was primarily performed using a 45° curved suture hook (Linvatec, Largo, FL, USA) loaded with absorbable 0# PDS sutures (Ethicon, Somerville, NJ, USA), supplemented by the inside-out repair technique using non-absorbable braided suture with the needle (ConMed Corporation, USA) when indicated.

### Rehabilitation protocol

Patients in both groups followed an identical postoperative rehabilitation protocol. Progressive rehabilitation begins immediately after surgery. A hinged knee brace is used for the first 4 weeks. The brace can be unlocked, allowing passive range of motion exercises from 0° to 90°. The knee joint should achieve full extension (0°), but hyperextension should be avoided. Partial weight-bearing starts 4 weeks postoperatively, followed by full-weight-bearing walking at 6 weeks. Gradual return to preoperative sports activities is expected within 9 to 12 months after surgery.

### Outcomes measures

#### Primary outcomes

The primary outcome measure was the difference in the 2-year healing rate of longitudinal and bucket-handle tears in the meniscus posterior horn between osteotomy and non-osteotomy patients. Meniscal healing status was assessed by MRI and second-look arthroscopy at final follow-up, and classified into three categories: complete healing, partial healing, and non-healing. All patients underwent a second surgery for hardware removal, during which a second-look arthroscopy was routinely performed. The final healing status was determined under direct arthroscopic visualization (sometimes with the aid of a probe).

#### Secondary outcomes

The secondary outcome measure of this study was to determine the association between meniscus posterior horn healing status and clinical outcomes. Clinical outcomes included: (1) Physical examination: Lachman test, KT-1000 side-to-side difference (SSD, KT-1000 arthrometer), pivot-shift grade under anesthesia [negative, 1+ (glide), 2+ (clunk), or 3+ (locking)]; (2) Second-look arthroscopic findings: cartilage damage, graft laxity, graft failure. In this study, patients who had any of the following features at the final follow-up were confirmed to have graft laxity: (1) KT-1000 SSD > 3 mm or positive pivot-shift test under anesthesia, or (2) graft laxity with probing on second-look arthroscopy. Graft failure was defined as complete discontinuity of graft fibers observed on follow-up MRI scans or second-look arthroscopy. Detailed methods for measurement acquisition were described by Wang et al. [18].

### Sample size

Due to the limited cohort of patients presenting with combined medial/lateral bucket-handle or longitudinal meniscal tears involving posterior horns who underwent concomitant osteotomy, this study comprehensively evaluated all combined osteotomy cases from 2016 to 2023. Because some cases involved concurrent tears of both the medial and lateral menisci, we included all patients with meniscal tears in this study to minimize sampling error. A post-hoc power analysis (GPower 3.1) was conducted based on predetermined parameters: all cases (osteotomy vs. control: 20 vs. 48) with α = 0.05. Using an effect size of 0.55 for chi-square testing of healing rates of medial MPHTs, the calculated statistical power (1−β) reached 0.96.

### Statistical methods

All quantitative data were tested for normality. Normally distributed quantitative data were recorded as mean ± standard deviation (SD), while non-normally distributed data were recorded as median (interquartile range, IQR). Categorical variables were presented as frequencies (%). The differences in quantitative variables between groups were analyzed using t-tests or Wilcoxon rank-sum tests, while differences in categorical variables were analyzed using Chi-square tests or Fisher’s exact tests. All statistical analyses were performed using the SPSS software (Version 26.0; IBM). The significance level was set at 0.05.

## Results

A total of 68 patients were included in the analysis, with 20 cases in the osteotomy group. The mean age of the patients was 30.3 years, and 73.5% were male. The average PTS was 17.5° (15° to 21°), and the anterior tibial subluxation in the lateral compartment was 7.9 mm (6.0 mm to 12.2 mm). Both groups were followed up for more than 2 years, with a mean follow-up duration of 2.9 years. The two groups demonstrated good comparability in terms of age (osteotomy vs. control: 30.3yrs vs. 31.2yrs), sex (male in osteotomy vs. control: 73.5% vs. 70%), time from injury to surgery (TFIS) (osteotomy vs. control: 20weeks vs. 24weeks), preoperative KT-1000 side-to-side difference (SSD) (osteotomy vs. control: 8.2 mm vs. 9.0 mm), ATS (osteotomy vs. control: 7.9 mm vs. 8.2 mm), and graft diameter (osteotomy vs. control: 9.1 mm vs. 8.9 mm) (all *p* > 0.05) (Table 1).

**Table 1:** Characteristics of patients with MPHTs between the two groups

Variables	Total (*n* = 68)	Osteotomy (*n* = 20)	Control (*n* = 48)	*P* value
Age (years)	30.3 ± 8.0	31.2 ± 6.6	29.9 ± 8.6	0.557
Sex (male)	50 (73.5%)	14 (70%)	36 (75%)	0.670
Side (left)	33 (48.5%)	8 (40%)	25 (52.1%)	0.364
BMI (kg/m^2^)	24.2 ± 3.2	24.7 ± 2.4	24.0 ± 3.5	0.319
TFIS (weeks)	20 (2, 48)	24 (2, 48)	18 (2, 48)	0.659*
KT-1000 SSD (mm)	8.2 ± 2.7	9.0 ± 3.1	7.9 ± 2.5	0.115
PST (1+)	16 (23.5%)	3 (15%)	13 (27.1%)	0.413
2+	48 (70.6%)	15 (75%)	33 (68.8%)	
3+	4 (5.9%)	2 (10%)	2 (4.2%)	
Lachman test (I grade)	19 (27.9%)	4 (20%)	15 (31.3%)	0.362
II grade	38 (55.9%)	14 (70%)	24 (50%)	
III grade	11 (16.2%)	2 (10%)	9 (18.8%)	
PTS (deg)	17.5 ± 1.7	18.2 ± 1.9	17.3 ± 1.6	0.053
ASLC (mm)	7.9 ± 2.0	8.2 ± 2.5	7.8 ± 1.7	0.486
ASMC (mm)	4.4 ± 3.2	5.2 ± 3.1	4.1 ± 3.2	0.231
Graft diameter (mm)	9.1 ± 0.9	8.9 ± 0.7	9.2 ± 0.9	0.083
Follow-up (years)	2.9 ± 0.5	2.8 ± 0.5	2.9 ± 0.6	0.614

BMI, body mass index; TFIS, time from injury to surgery; KT-1000 SSD, KT-1000 arthrometer side-to-side difference; PTS, posterior tibial slope; PST, pivot shift test; ASLC, anterior subluxation of the lateral compartment; ASMC, anterior subluxation of the medial compartment; *Mann-Whitney U Test.

### Characteristics of meniscus posterior horn in both groups

#### Medial MPHTs

**Fig. 4:**
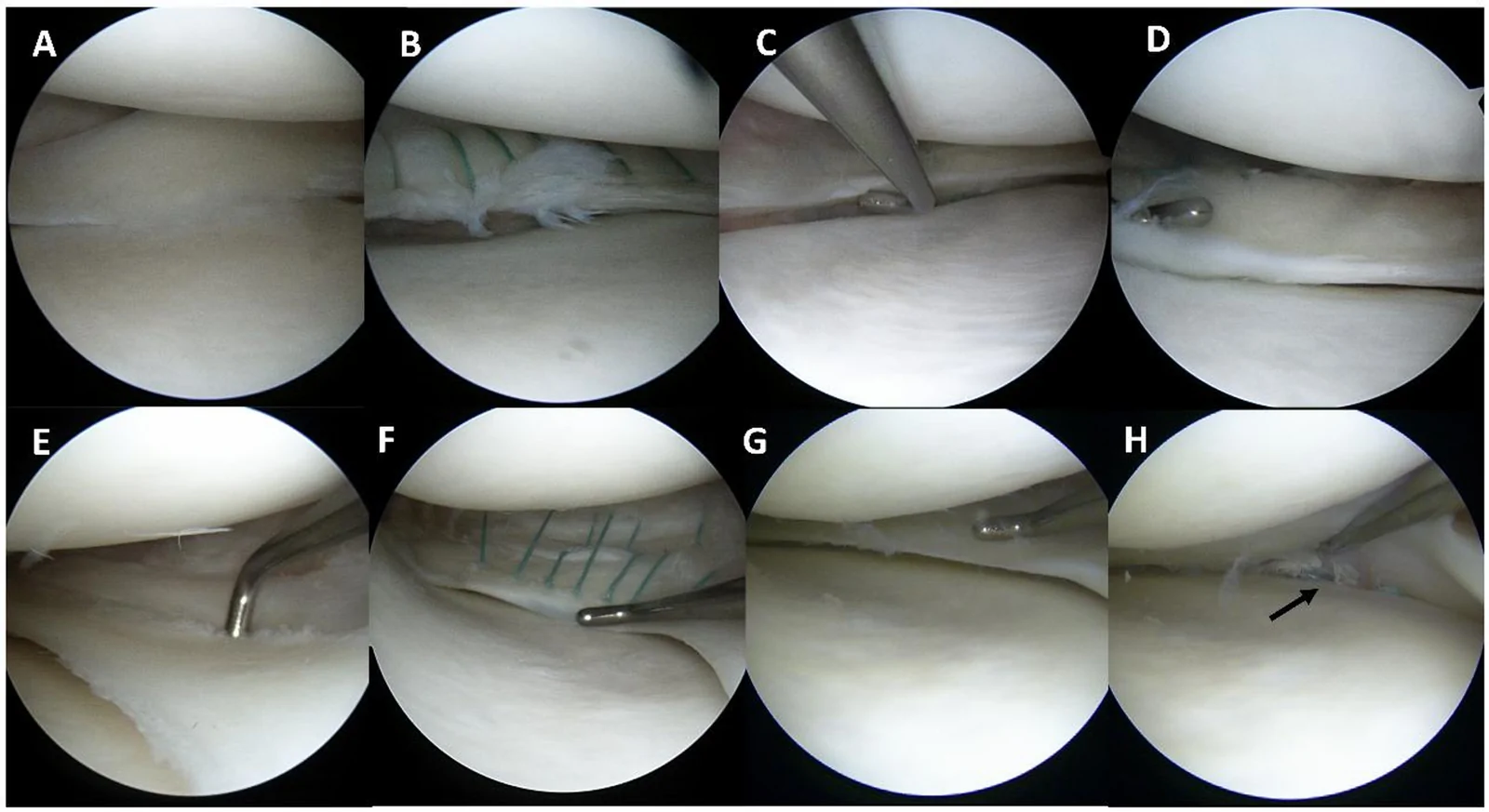
Arthroscopic findings in two cases of longitudinal tears involving the posterior horn of the medial meniscus, showing preoperative **A** and **E**, intraoperative **B** and **F**, and final follow-up **C**–**D** & **G**–**H** stages. **A** & **E** Longitudinal tears involving the medial posterior horn; **B** & **F** Inside-out suture technique performed for repair; **C**–**D** Solid healing achieved on both superior and inferior surfaces of the posterior horn; **G** Complete healing of the superior surface of the posterior horn; **H** Partial healing of the inferior surface of the posterior horn

In the osteotomy group, 17 patients had meniscus tears (11 longitudinal + 6 bucket-handle tears). At the 2-year follow-up, all bucket-handle tears in MPHTs achieved complete healing. Among the longitudinal tears, 1 showed partial healing in the posterior horn. In the control group, 34 patients had meniscus tears (23 longitudinal + 11 bucket-handle tears). At the final follow-up, 8 longitudinal and 2 bucket-handle tears showed partial healing in MPHTs, while the remaining cases achieved complete healing (Table 2). The repaired meniscus body and anterior horn portions have both achieved solid healing. Figure 4 demonstrated the different healing status under arthroscopy following suture repair in two cases of longitudinal tears involving the posterior horn of the medial meniscus.

#### Lateral MPHTs

In the osteotomy group, 10 patients had concomitant meniscus tears (6 bucket-handle + 4 longitudinal tears). In the control group, 33 patients exhibited meniscal tears (20 longitudinal + 13 bucket-handle tears). At the final follow-up, 1 bucket-handle tear in the osteotomy group showed partial healing, whereas in the control group, 4 longitudinal and 1 bucket-handle tear demonstrated partial healing in lateral MPHTs, with the remaining tears achieving complete healing (Table 2). Both the repaired meniscus body and anterior horn portions have healed firmly.

### Healing rate comparison

Pooled data analysis revealed a statistically significant improvement in healing rates in medial MPHTs in the osteotomy group versus controls (94.1% vs. 70.6%, *p* = 0.036). In contrast, concomitant osteotomy procedures did not significantly affect the healing outcomes in lateral MPHT repairs (90% vs. 84.8%, *p* = 0.680) (Table 3).

**Table 2:** Basic info on meniscus tears and healing features of meniscus posterior horns in both groups

Osteotomy group (*n* = 17)	No. (%)	Control group (*n* = 34)	No. (%)
*Medial meniscus*			
Longitudinal tear	11	Longitudinal tear	23
Complete healing	10 (91)	Complete healing	15 (65)
Partial healing	1 (9)	Partial healing	8 (35)
Non-healing	0	Non-healing	0
Bucket-handle tear	6	Bucket-handle tear	11
Complete healing	6 (100)	Complete healing	9 (82)
Partial healing	0	Partial healing	2 (18)
Non-healing	0	Non-healing	0

Osteotomy group (*n* = 10)	No. (%)	Control group (*n* = 33)	No. (%)
*Lateral meniscus*			
Bucket-handle tear	6	Longitudinal tear	20
Complete healing	5 (83)	Complete healing	16 (80)
Partial healing	1 (17)	Partial healing	4 (20)
Non-healing	0	Non-healing	0
Longitudinal tear	4	Bucket-handle tear	13
Complete healing	4 (100)	Complete healing	12 (92)
Partial healing	0	Partial healing	1 (8)
Non-healing	0	Non-healing	0

MPHTs, meniscal posterior horn tears.

**Table 3:** The comparison of the healing rate of the meniscus posterior horns between groups

	Osteotomy group	Control group	*P*-value
Medial MPHTs	16/17 (94.1%)	24/34 (70.6%)	0.036
Lateral MPHTs	9/10 (90%)	28/33 (84.8%)	0.680

MPHTs, meniscal posterior horn tears.

### Association between partial healing and graft outcomes

Among patients who did not undergo PTS correction, partial healing of the medial MPHTs, as compared with complete healing, was associated with residual graft laxity (50% vs. 16.7%, *p* = 0.045), but not with a positive pivot shift (1+/2+, 40% vs. 12.5%, *p* = 0.071) (Table 4).

**Table 4:** Comparisons of clinical outcomes between two healing statuses of medial MPHTs in the control group

	Healing (*n* = 24)	Partial healing (*n* = 10)	*P*-value
KT-1000 SSD (mm)	1.7 + 2.8	3.0 + 3.2	0.244
*Pivot grade*			
Negative	21 (87.5%)	6 (60%)	0.071
1+/2+	3 (12.5%)	4 (40%)	
Lachman test (negative)	19 (79.2%)	7 (70%)	0.566
I grade	5 (20.8%)	3 (30%)	
Cartilage injury (I-II grade)	1 (4.2%)	2 (20%)	0.160*
Graft laxity (yes)	4 (16.7%)	5 (50%)	**0.045**
Graft failure (yes)	3 (12.5%)	2 (20%)	0.583

KT-1000 SSD, KT-1000 arthrometer side-to-side difference; *Fisher’s exact test.

## Discussion

The most important findings of this study can be summarized as follows. For ACL-injured patients with a steep posterior tibial slope, excessive anterior tibial subluxation, and concomitant long longitudinal or bucket-handle tears involving the posterior meniscal horn, concomitant slope-reducing tibial osteotomy was associated with a higher healing rate of the repaired posterior horn of the medial meniscus. In knees without slope correction, incomplete healing of the posterior horn of the medial meniscus may be associated with residual ACL graft laxity.

Studies have demonstrated that with accurate diagnosis and timely repair of posterior meniscal horn tears, knee joint loading conditions can be restored to near-normal levels [21], significantly slowing the progression of osteoarthritis [22, 23], particularly in patients with multi-ligament knee injuries and concomitant ACL tears. Previous studies have reported that meniscal horn tears occur in 18% of chronic ACL-deficient knees and 12% of acute ACL injuries [1, 2]. Matheny et al. analyzed 50 patients with posterior meniscal horn tears, among whom 60% had concomitant ACL injuries [24]. Further analysis revealed that patients with lateral MPHTs were significantly more likely to have ACL tears (OR = 10), while medial MPHTs showed a stronger association with chondral defects (OR = 6). Although clinical evidence suggests that meniscal repair demonstrates enhanced healing rates when performed concomitantly with anterior cruciate ligament reconstruction [25, 26] (potentially mediated by growth factor release [27]), the assessment of postoperative healing capacity for repaired posterior meniscal horn tears remains inadequate in patients with anatomic risk factors, such as steep PTS and excessive ATS.

In ACL reconstruction, the failure rate of repaired medial meniscus longitudinal tears or bucket-handle tears can be as high as 20% [28]. Apart from surgical techniques, identifying and addressing the anatomical risk factors that affect the peri-meniscal mechanical environment may promote healing of the repaired meniscus. A recent systematic review involving 1313 patients (10 studies, 884 with MPHTs vs. 429 controls) found that patients with lateral MPHTs had a greater lateral PTS (7.3° vs. 5.7°), while those with medial MPHTs exhibited a more pronounced medial PTS (2.3° vs. 1.3°) [5]. Similarly, Bernholt et al. reported that ACL-injured patients with lateral MPHTs had higher medial and lateral PTS [9]. These findings suggest that posterior meniscus horn tears are significantly associated with increased PTS, regardless of concomitant ACL injury. A recent cadaveric study has also demonstrated that an increased PTS increases the force on the posterior meniscus horn [10]. This indicates that the rise in compressive and posterior shear forces during knee loading may subject the posterior meniscus horn to greater stress, increasing the risk of failure after repair. From a clinical follow-up comparison perspective, this study suggests that slope correction was associated with the healing rate of repaired posterior meniscus horns. Our findings provide clinicians with an additional repair strategy when managing such complex cases, aiming to optimize the biomechanical environment of the posterior meniscus horn and enhance healing outcomes. However, due to the retrospective non-randomized design, causation cannot be inferred, our findings only support an association.

Current evidence suggests that slope-reducing tibial osteotomy combined with ligament reconstruction represents a reliable indication for failed ACL reconstruction cases with increased PTS, particularly in those with PTS > 12° [29]. However, whether this osteotomy procedure should be applied during primary ACLR remains controversial. Wang et al. recently demonstrated that concomitant osteotomy effectively reduces postoperative ATS and graft laxity in primary ACLR patients, with greater clinical benefits observed in cases exhibiting higher PTS angles [18]. Their proposed 16° (measured with full-length lower limb radiography) is the optimal threshold for osteotomy in primary ACLR. Biomechanical studies indicate that increased PTS increases anteroposterior shear stress on the meniscus, potentially exacerbating ATS [10, 30]. This supports the notion that steep PTS serves as a shared risk factor for ACL tears, excessive ATS, and meniscal horn tears. Therefore, for primary ACL injuries combined with steep PTS, excessive ATS, and MPHTs, slope-reducing tibial osteotomy may represent a more reliable treatment option.

This study also investigated the impact of incomplete healing of the posterior horn of the medial meniscus on knee stability and ACL graft outcomes in patients without slope correction in an exploratory fashion. The findings suggested that incomplete healing of the posterior horn may be associated with residual ACL graft laxity. Previously, Malinowski et al. [31] reported that among 9 cases showing incomplete meniscal healing during early second-look arthroscopy (6–8 weeks post-primary surgery), 6 cases (66.7%) achieved clinical success at 2-year follow-up. Although early incomplete meniscal healing may not necessarily indicate repair failure, their study did not specifically evaluate knee stability parameters and graft outcomes in these cases [31]. The relationship between incomplete meniscal healing and long-term clinical outcomes requires further evaluation. In summary, this study emphasizes the critical importance of incomplete healing of the posterior meniscal horn. In patients with multiple anatomic risk factors, such as steep PTS and excessive ATS, consideration should be given to more aggressive surgical interventions.

This study has limitations. First, postoperative subjective functional assessments were not evaluated, limiting comparative analysis of patient-reported outcomes. Second, the radiographic visibility of osteotomy precluded the blinding of assessors to surgical groups. Third, the retrospective design may have introduced inherent patient selection biases. Fourth, despite including all eligible osteotomy cases, the final cohort remained relatively small, potentially affecting statistical power. Fifth, the follow-up period was insufficient to determine the long-term impact of slope correction on the healing rates of repaired posterior meniscal horn tears. Finally, due to limitations related to sample size and confounding variables, regression analysis was not performed in this study to investigate the relationship between osteotomy and meniscal healing status.

## Conclusion

For ACL-injured patients with a steep posterior tibial slope (≥ 15°), an excessive anterior tibial subluxation (≥ 6 mm), and concomitant long longitudinal or bucket-handle tears involving the posterior meniscal horn, concomitant SRTO was associated with a higher healing rate of the repaired posterior horn of the medial meniscus. In knees without slope correction, incomplete healing of the posterior horn of the medial meniscus may be associated with residual ACL graft laxity.

## Data Availability

Data yielded in our study will be made available by the authors to any qualified researchers.

## Abbreviations

ACLR: Anterior cruciate ligament reconstruction
SRTO: Slope-reducing tibial osteotomy
PTS: Posterior tibial slope
MPHTs: Meniscal posterior horn tears
ATS: Anterior tibial subluxation
ASLC: Anterior subluxation of the lateral compartment
ASMC: Anterior subluxation of the medial compartment
TFIS: Time from injury to surgery
IQR: Interquartile range
SSD: Side-to-side difference

## Code availability

Yes.
